# Heavy-Metal Speciation Distribution and Adsorption Characteristics of Cr (VI) in the Soil within Sewage Irrigation Areas

**DOI:** 10.3390/ijerph19106309

**Published:** 2022-05-23

**Authors:** Songtao Liu, Furong Yu, Jianuo Zhang

**Affiliations:** 1 College of Geosciences and Engineering, North China University of Water Resources and Electric Power, Zhengzhou 450046, China; st.liu@stu.ncwu.edu.cn (S.L.); zhang@stu.ncwu.edu.cn (J.Z.); 2 Collaborative Innovation Center for Efficient Utilization of Water Resources, Zhengzhou 450046, China; 3 Key Laboratory of Water and Soil Resources Conservation and Restoration in the Middle and Lower Reaches of Yellow River Basin, Ministry of Natural Resources, Zhengzhou 450046, China

**Keywords:** heavy metals, soil, sediment, speciation distribution, adsorption characteristics, sewage irrigation

## Abstract

While sewage irrigation relieves water shortages in Northern China, its excessive application triggers a series of environmental problems, such as heavy-metal pollution. Soil profile and river sediment profile samples from the sewage irrigation area (SIA) were collected by selecting the farmlands in which sewage irrigation activity has been reported since the 1960s, around Huiji River (HJR) and Huafei River (HFR) in Kaifeng, Henan Province, China, as research areas. In this study, the total amount of heavy metals (Cr, Cd, Pb, Mn, Zn, and Ni) and the heavy-metal speciation analysis using the modified BCR sequential extraction method were used to evaluate the impacts of wastewater on agricultural soils and the potential risk. Furthermore, the least contaminated Cr (VI) was selected for the study of adsorption characteristics to determine the environmental capacity of soils for heavy metals when the composition of wastewater changes under long-term effluent irrigation conditions. The results show that: (1) the concentrations of heavy metals in soil continuously decreased with depth, while the opposite was observed in sediment, reflecting the continuous improvement in water quality over the historical period; (2) In the topsoil, the mean concentrations (mg·kg^−1^) in rank order are as follows: Mn (588.68) > Zn (284.21) > Pb (99.76) > Cr (76.84) > Ni (34.71) > Cd (3.25), where Cd exceeded the control value by 3.15 times around HFR, and sediment samples also showed higher heavy metal concentrations in HFR than in HJR; (3) Speciation distribution and risk assessment code (RAC) indicate that Mn and Cd were at medium risk and that Cd warrants attention due to its being a non-essential toxic element in humans; (4) The adsorption rates of soil in various layers in different profiles within SIAs for Cr (VI) gradually increased with the increasing initial content of Cr (VI). Among the three isothermal adsorption models, the fit result obtained by the Langmuir equation was superior to those obtained by the Freundlich equation and the linear equation.

## 1. Introduction

In many agricultural activities, river water containing untreated or incompletely treated industrial and domestic wastewater has to be utilized to irrigate crops due to limited freshwater resources, especially in some relatively undeveloped areas [1]. Nearly 70% of the world’s freshwater resources are used for agricultural irrigation [2], and such sewage irrigation has undoubtedly mitigated water shortages in many arid and semi-arid regions, and has even become a common practice in some Mediterranean countries, such as Spain, Greece, and Egypt [3,4]. The North China Plain is an important grain-growing area in China, although it possesses a semi-humid climate and is still perennially arid or semi-arid climate in individual places. Coupled with the excessive use of groundwater and surface water receiving multiple pollutants for a long period of time, this makes part of the agricultural area reliant on long-term sewage irrigation. Some studies indicate that the nutrients (such as nitrogen, phosphorus, and potassium) and metals in wastewater are absorbed by plants to help increase yield, which improves soil properties and is beneficial to agricultural production [5,6,7]. However, some unnecessary or toxic heavy metals that are simultaneously present in wastewater can aggravate the deterioration of soil structure and properties, resulting in soil contamination, and potentially endangering human health through crop uptake (e.g., Cr, Cd, Pb, Ni, etc.) [8,9,10]. Even the essential trace heavy metals (e.g., Fe, Mn, Zn, etc.) required for plant growth can become toxic when they accumulate beyond the tolerance threshold [11,12].

Several studies have shown that the edible parts of various plants exert different absorption effects on heavy metals in the soil [1,4,13,14,15]. The uptake of non-essential metals such as Cd, Pb, and Hg can inevitably lead to health hazards during human consumption; even if the ingestion is relatively low, long-term consumption will accumulate due to the bio-accumulation of heavy metals [16,17,18]. The total amount of heavy metals is usually used as a critical factor to assess the contamination status of soil, but this is gradually being considered unreliable, especially in terms of bio-accessibility [19]. As a result, several methods have been developed for the study of chemical form and bioavailability [19,20,21], among which the BCR sequential extraction method aims to further classify heavy metals according to their release potential under altered conditions in the surrounding environment, and some evaluation methods are derived [22]. Using the BCR sequential extraction method, a selective and progressively stronger extractant is being used to successively dissolve each heavy metal in each extraction step according to its release characteristics; ultimately, each heavy metal was experimentally classified into exchangeable, reducible, oxidizable, and residual states [23]. Compared with another widely adopted Tessier sequential extraction method, the BCR protocol requires a longer reaction time but simplifies many steps, e.g., the exchangeable states (extracted with magnesium chloride) and the carbonate-bound state (extracted with sodium acetate) in Tessier’s protocol are combined into the exchangeable states (extracted with ammonium acetate) in BCR, while the weak and strong organic states are considered oxidizable states in BCR [24]. In response to the time-consuming problem of the BCR protocol, some scholars introduced techniques such as ultrasonic shock, microwave and continuous flow into the experimental process, which significantly improved the extraction efficiency [25,26,27,28]. Usually, the exchangeable state refers to clay minerals adsorbed in the soil, as well as other components, such as heavy metals on iron hydroxide, manganese hydroxide, and humus, which are the most likely to migrate and transform in the environment and enter the food chain. The water-soluble state is classified in the exchangeable state because its content is often below the detection limit, while the residual state is the most stable due to it representing most of the primary and secondary minerals present in the crystalline lattice [24,29]. The reducible fraction is the iron and manganese oxides attached to the surface of the particles, which will reduce the thermodynamic stability under anoxic conditions, and are usually extracted using hydroxylamine hydrochloride as the reducing agent [19,29]. The oxidizable state refers to the heavy metals in the particulate matter in different forms, entering or wrapped around the organic matter particles with organic-matter-chelated or -generated sulfides. Under oxidation conditions with heavy metals, those combined with organic materials are easy-to-release heavy metals, while the oxidation process usually also oxidizes metal sulfides, so the organic state in the Tessier’s protocol is considered the oxidizable part [29].

Moreover, soil’s adsorption characteristics for heavy metals are also important factors in determining plants’ uptake and contamination [30]. Plants’ ability to absorb nutrients differs between soil types, depending on the soil properties [31]. Cr (VI) is a non-essential element for humans and is more toxic and transportable than Cr (III) [32,33]. There is a clear consensus that plants are more susceptible to Cr (VI) uptake from the soil and being consumed by humans through this pathway, as reported in some earlier studies [34,35,36]. In this study, the soil samples from the sewage irrigation area (SIA) were collected for adsorption experiments to explore the adsorption characteristics of Cr (VI) and evaluate the migration capacity of Cr (VI) as toxic under the changing conditions of the surrounding wastewater.

Many studies in SIA focused on the total concentration of heavy metals and the assessment of the pollution status, or on the growth characteristics in different crops under the influence of heavy metals [37,38,39,40]. As a typical long-term SIA in the North China Plain, some studies in Kaifeng have shown that industrial activities in the SIA caused different degrees of heavy-metal pollution in rivers and soils, among which Cd pollution is significant [41,42]. Furthermore, a study found that the enrichment of Cd and As in wheat seeds grown in the Kaifeng, China exceeded the maximum acceptable risk levels recommended from by the U.S. Environmental Protection Agency (EPA) and International Radiation Protection Association (IRPA), which could cause harm to humans via the dietary route [43]. In this study, by collecting soil and river sediment profile samples, a more insightful speciation analysis and isothermal adsorption experiments were conducted to estimate the release potential of heavy metals and their adsorption characteristics. The results, combined with previous studies on the selective uptake of plants, can provide a basis for the improvement and utilization of agricultural soils under the influence of long-term effluent irrigation.

## 2. Materials and Methods

### 2.1. Study Area

A representative farmland area with a history of more than 50 years of sewage irrigation in southeastern Kaifeng was selected, which was located between two medium-sized towns and near the confluence of two rivers, the Huiji River (HJR) and the Huafei River (HFR). As a branch of HJR, HFR receives industrial wastewater discharged from various enterprises (including a fertilizer factory, zinc refinery, pharmaceutical factory, and leather factory) in Kaifeng situated around the river. The wastewater discharged into HFR is still rich in many heavy metals, even if it is subjected to preliminary treatment, owing to the complexity, diversity and multiplicity of surrounding improperly discharging manufacturing enterprises [44].

Kaifeng sits in the central part of Henan Province, China, which has a sub-humid, semi-arid continental monsoon climate. The study area has an annual average temperature of 14.1 °C and annual average precipitation of 586.3 to 628.0 mm [45]. Precipitation mainly occurs in June, July, and August, accounting for about 57.8% of the annual precipitation. A windy, rainless, and dry climate in winter and spring is characteristic of the study area, and evaporation accounts for 69% of the annual precipitation. The research area is at an altitude of 68 m, where soil appears as loam or sandy loam in the alluvial plain of the Yellow River.

### 2.2. Sampling and Sample Pretreatment

**Figure 1:**
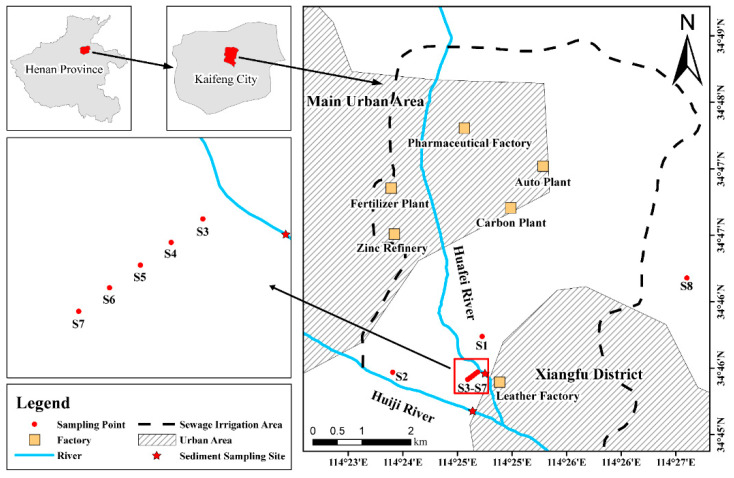
Positions of sampling points.

The unsaturated zones of the farmland soil around the sampling points mainly contain silty soil and clay, which are widely distributed therein. According to the local situation, sample collection was conducted in June 2020, and the sampling was divided into profile sampling and cross-section sampling, as well as river sediment samples. Seven farmland (S1–S7, n = 18) and two sediment (n = 15) sampling sites and one control site (S8, n = 5) were collected for subsequent testing, leading to a total of 38 profile soil samples. The research areas and sampling points are shown in Figure 1, where S3–S7 are cross-sectional sampling points situated at 50-m intervals along the HFR, and river sediment sampling sites in the HFR and HJR, respectively. To ensure accuracy in experimental sample collection, S8, which is 1 km east of the SIAs, was selected as the contrast site for this study. The soil and sediment profile samples were sampled at depths ranging from 0 to 900 mm and 0 to 600 mm, respectively, and divided into 100-mm intervals. A column sampler was adopted to collect the sediment samples which were encapsulated with polyethylene spatulas and plastic zip-lock bags, then transported at 4 °C for subsequent experiments. Equipment during collection was previously soaked overnight in 10% (V:V) nitric acid solution and rinsed with deionized water. After the soil and sediment samples were freeze-dried, various impurities (such as residual roots and stones) were removed, then ground and sieved to the size < 74 mm (200-mesh screen) because the heavy metals are mostly enriched in finer particles. To ensure the stability of the samples, the sieved samples were re-encapsulated in smaller plastic zip-lock bags and stored at −10 °C until the next experiment.

### 2.3. Chemical Analysis

#### 2.3.1. Pseudo-Total Concentrations of Heavy Metals

An inductively coupled plasma mass spectrometer (ICP-MS, Agilent Technologies 7800, Santa Clara, CA, USA) was used to measure the Cr, Cd, Pb, Mn, Zn, and Ni contents of the samples. To comply with the requirements of the liquid sample injection, the HNO_3_-HF-HCl system was used for the digestion method of the soil and sediment samples. The digestion process was described as follows: (1) 0.2 g of each soil sample was weighed and transferred into a Teflon digestion tube, then a small amount of deionized water was added to avoid a violent reaction between the acid solution and the sample; (2) After gently shaking to mix the sample and deionized water, nitric acid (68.0%, 15.3 mol·L^−1^), hydrofluoric acid (99.9%, 33.3 mol·L^−1^), and hydrochloric acid (37.0%, 12 mol·L^−1^) were slowly added to the Teflon tube in the sequence of 6 mL, 2 mL, and 2 mL, respectively; (3) After another slight mixing, the samples were digested using an automatic microwave digester (Sineo MDS-6G); (4) Due to the possibility of corroding the ICP-MS with excessive acid, the majority of the residual acid reaction solution was removed by an electro-thermal plate after the digestion process was completed, until the residue appeared in a colloidal state. The residual sample was diluted and filtered by the nitrocellulose membrane syringe filter (0.45 μm), and the metals content was measured by ICP-MS. The detection limits of the Cr, Cd, Pb, Mn, Zn, and Ni were Cr (0.08 μg·L^−1^), Cd (0.4 μg·L^−1^), Pb (0.3 μg·L^−1^), Mn (0.2 μg·L^−1^), Zn (5 μg·L^−1^), and Ni (0.1 μg·L^−1^), respectively.

#### 2.3.2. Analysis of Heavy-Metal Speciation

A modified BCR sequential extraction method was used to measure the speciation of heavy metals in samples. The experimental procedure was based on the European Community Bureau of Reference, and each heavy metal was classified into exchangeable fraction (F1, soluble species, cation exchange sites and bound to carbonates), reducible fraction (F2, bound to Fe and Mn oxides), oxidizable fraction (F3, bound to organic matter and sulfides) and residual fractions (F4, bound to the mineral matrix) according to the release characteristics reflected in different environments [23]. Referring to related studies, the amount of sample and extractant in the initial modified BCR extraction protocol was reduced in equal proportions [46]. This extraction scheme with standard substances (GBW07436, standard substances for the sequential extraction of heavy metals in lake sediments) was verified in our experiments and the results found that it had no effect on the results and was able to significantly reduce the evaporation time in the third step (F3) and improve the mixing effect in each step.

A total of 0.8 g of freeze-dried and sieved sample was placed in a 50-mL polyethylene centrifuge tube for sequential extraction experiments, using the extractant and concentrations listed in Table 1. As mentioned in the BCR protocol [23], the supernatant was extracted and filtered (0.45 μm) for testing by centrifugation for 20 min at 4000 rpm after each step, and washed by shaking with 15 mL of deionized water for 15 min, then centrifuged again and the wash solution poured off. Guaranteed reagent-grade nitric acid was used to adjust the pH value of the extractant solution to the values listed in Table 1.

#### 2.3.3. Quality Control and Data Analysis

The reagents used in the experiments were of analytical reagent grade and above, and the resistivity of deionized water (Milli-Q Millipore) was greater than 18 MΩ·cm^−1^. All chemical analysis work was performed in the key laboratory of water and soil resources conservation and restoration (Ministry of Natural Resources of China).

A total of 20% of the samples were randomly selected as parallel samples for three times during digestion procedure and tested by ICP-MS, and the analytical results were calibrated by reagent blanks and national standard soil samples (GBW07401, geophysical standard reference sample soil) to ensure that the standard deviation was less than 15%. To ensure accuracy during sample testing, the internal standard method recommended by Agilent 7800 was used and a deionized water specimen was tested every 12 samples to verify the residual elements in the instrument injection channel. Likewise, the reagent blanks and parallel samples were used for quality control of the experimental procedure during the process of BCR sequential extraction, and the results were calibrated by national standard soil samples (GBW07436, standard substances for sequential extraction of heavy metals in lake sediments). Finally, an internal check was conducted on the results of the BCR sequential extraction by comparing the total amount of extracted fractions with the pseudo-total concentrations of heavy metals by digestion [47,48]. Recovery of the BCR sequential extraction method was calculated by the following formula in Equation (1):

Recovery = [(C_F1_ + C_F2_ + C_F3_ + C_F4_)/C_Total digestion_] × 100%, (1)

where C represents the different fractions and the total measured concentration of heavy metals. The sum of each fraction of each heavy metal in the sample was calculated to ensure a recovery that was maintained between 85% and 115%.

Analysis of variance (ANOVA) with LSD and 95% and 99% confidence levels could be used to estimate the differences between samples by IBM-SPSS statistical software (Version 22.0) (International Business Machines Corporation, Armonk, NY, USA) (*p* < 0.05 or *p* < 0.01).

### 2.4. Potential Risk Assessment of Heavy Metals

The risk assessment code (RAC) was used to assess the potential risk of heavy metals in soil samples within the SIA. The RAC method allows for the direct evaluation of the environmental response based on the proportion of exchangeable fraction (F1) in heavy metals due to its weak binding to particulates; this results in the exchangeable fraction (F1) being more susceptible to migration and enrichment in the food chain [49], as given by Equation (2):

RAC = [C_F1_/(C_F1_ + C_F2_ + C_F3_ + C_F4_)] × 100%, (2)

where C_F1_, C_F2_, C_F3_, and C_F4_ are the contents of the exchangeable fraction, reducible fraction, oxidizable fraction and residual fraction, respectively. The RAC index can be categorized into five classes (Table 2) based on initial research experience and the results of numerous studies [49,50,51].

### 2.5. Adsorption Characteristics of Soil for Cr (VI)

By conducting a batch test, 10 soil samples (10 g for each) from each of the profiles S1 and S2 were weighed and separately placed into a 100-mL beaker sealed with a ground-glass stopper; then, 50 mL of Cr (VI) solutions with different concentrations were separately added to the beakers. By taking NaNO_3_ with a concentration of 0.01 mol·L^−1^ as the supporting electrolyte, the concentrations of Cr (VI) solutions were adjusted to 10, 20, 30, 50, 80, 120, 200, 500, 900, and 1200 mol·L^−1^ and the pH was adjusted to 6; subsequently, the beakers were placed into a constant-temperature oscillator shakes at 145 rpm for three hours at 25 °C ± 0.5 °C. After standing, the supernatants were separately taken, centrifuged, and filtered. The concentration of Cr (VI) in filter liquors was measured using flame atomic absorption spectrometer (FAAS, ZEEnit700P, Analytik Jena, Jena, Germany); then, the adsorption capacities and adsorption rates were calculated. Each treatment was conducted in triplicate. Isothermal adsorption experimental tests were performed at North China University of Water Resources and Electric Power.

## 3. Results and Discussion

### 3.1. Vertical Distribution of Heavy Metals

Soil profile samples were collected along the HFR (S1) and HJR (S2) in the SIA, and as control sites, a set of profile samples were selected outside the SIA (S8); heavy metal data are listed in Table 3. The concentration (mg·kg^−1^) of heavy metals (Cr, Cd, Pb, Mn, Zn, and Ni) ranged from 47.7–69, 0.12–2.24, 18.1–59.9, 451.46–952.46, 62.18–256.68, and 16.46–32.13 in S1, respectively. Most of the heavy metals in S1 were at the highest concentration among the three sample sites, which is consistent with the various factories around the HFR. The concentrations (mg·kg^−1^) of heavy metals Cr, Cd, Pb, Mn, Zn, and Ni ranged from 58.6–80, 0.16–0.46, 22.1–64.3, 442.53–612.65, 66.2–184.05, and 17.28–25.75 in S2, respectively. Slightly higher concentrations of Cr and Pb were observed in the superficial soil at S2 compared to S1 (*p* > 0.05). As the concentrations of Cr (*p* > 0.05) and Pb (*p* < 0.01) in the sediment samples of the HFR were greater than those of the HJR, the slightly opposite variation in the profile samples probably appeared due to the selective uptake of soil elements by different crops, as shown in the study in which it is reported that the leaves of maize and the roots of wheat can accumulate Cr and Pb [52]. This may result in reductions in the Cr and Pb in the soil. Except for Cr, the control site had the lowest concentration (mg·kg^−1^) of heavy metals in the profile with Cr, Cd, Pb, Mn, Zn, and Ni in the ranges 73.4–91.1, 0.12–0.23, 18.8–30.7, 434.18–483.41, 62.67–97.81, and 16.49–20.11, respectively. The variation range data of heavy metals for the three profile samples indicate that Cr is in a relatively stable state, suggesting that Cr is assumed to be derived from the primary soil environment in the study area and is not caused by sewage irrigation. Whether Cr is considered a primary metal persisting in the soils of the study area will be determined based on the speciation analysis in Section 3.4.

**Figure 2:**
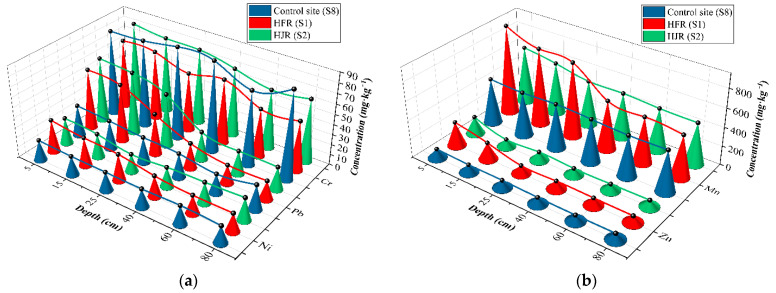
Concentrations of heavy metals in the soil profile (Ni, Pb, and Cr (**a**); Zn and Mn (**b**)).

In addition to the Cr, the concentrations of heavy metals decreased with increasing depth (Figure 2), which indicates the interception of heavy metals by the surface soil during irrigation, which corresponds to the findings of existing research [53]. Compared to the control site, the trend of decreasing heavy metal concentrations from the surface to greater depths in S1 and S2 is clearer, suggesting the accumulation of soil heavy metals by sewage irrigation and the adsorption of the surface soil to heavy metals in wastewater. Cd is not shown in Figure 2 due to the very low volumes thereof, but the standard deviation (SD) shows a large variation in S1 (0.82) and S2 (0.12), especially as the maximum value in S1 reaches 2.24 mg·kg^−1^. Significant decreasing trends and a large SD indicated that the heavy metals were enriched in the superficial soil in the SIA [54]. Moreover, in the deep soil, the heavy metal concentrations were similar to those at the control site (*p* > 0.05), indicating that heavy metals had not migrated downward from the wastewater under long-term sewage irrigation conditions, and crop uptake might be the main migration pathway.

### 3.2. Heavy Metal Concentrations in Sediment

**Figure 3:**
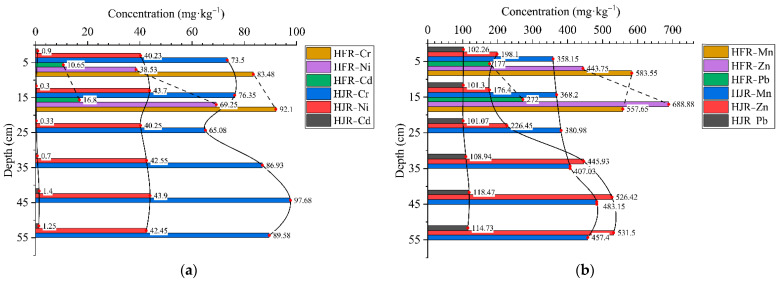
Heavy-metal concentrations of sediment profiles in the HFR and HJR (Cr, Ni, and Cd (**a**); Mn, Zn, and Pb (**b**)).

As indirect carriers of plant effluents around the study area, river sediments were collected and studied for heavy-metal concentrations (Figure 3). In geochronology, the short half-life isotopes ^210^Pb (t^1/2^ = 22.26 years) and ^137^Cs (t^1/2^ = 30.14 years) are commonly used to calculate the age of formation of sediment cores; with increasing depth, the sediment formation age gradually increases [55,56]. As a result, changes in heavy metal content in river sediment profiles could indicate long-term heavy metal-content changes in river water. In recent years, due to the reinforcements of the HFR channel, the river sediments were few, and only the surface sediments were collected (0–200 mm). In contrast, the HJR has deeper sediments, which were collected (0–600 mm) to provide more information about historical conditions in the river.

The increasing heavy-metal concentration with the sediment depth, and a slight decrease in Mn in HFR, indicate that significant metal content was once present in the river water and subsequently accumulated in the sediment. Referring to the sediment standards published by the Ontario’s Ministry of the Environment, the mean value of surface sediment (0–200 mm) far exceeded the standards for all heavy metals except for Cd in HJR, which, along with other wastewater catchment sediment data, are summarized in Table 4. Compared with other studies (Table 1), different extents of excess heavy metals in sediments are found in different areas due to their various anthropogenic impacts, such as the sediment of Mighan Lake and Masan Bay, which generally contains lower levels of heavy metals than river sediments, possibly due to the fact that rivers are more exposed to pollution sources. The heavy-metal contents in the HFR sediments were all higher than those in the HJR, with Cd being the most obvious. Cd is a non-essential element for humans, is considered harmful to humans and aquatic organisms, and is often used as an indicator of environmental pollution [57]. It is noteworthy that, in HJR sediment, the mean value and standard deviation of Cd were merely 0.81 mg·kg^−1^ and 0.42, respectively (Figure 3); this value was generally similar to those found in major river sediments in Beijing (0.42 mg·kg^−1^) [58] and Shanghai (2.69 mg·kg^−1^) [59], China. However, the high Cd content of the sediments in the HFR was 13.73 mg·kg^−1^ on average, which exceeded (by 137 times) the background values for sediments in mainland China (0.1 mg·kg^−1^) [60]. The significant difference between Cd content in the sediments suggests that the HFR received a large amount of inadequately treated industrial wastewater and that long-term irrigation with wastewater was an important reason for the high Cd content of agricultural soils near the HFR (Table 5). The study of the irrigation areas in north-west China also proved the relationship between heavy metals in sediments and agricultural soils [61]. There was no significant difference in Cr and Ni between the two river sediment samples (*p* > 0.05), but Ni was increased by 1.8 times at depths of 100–200 mm in HFR.

### 3.3. Heavy Metal Concentration in Surface Soil

Perpendicular to the HFR, five sections of cultivated topsoil samples (S3–S7) were collected between the two rivers and combined with the S1 and S2 topsoil data previously listed in Table 5. In the surface soil of the SIA, the concentrations (mg·kg^−1^) of Cr, Cd, Pb, Mn, Zn, and Ni had ranges of 54.9–97.2, 0.12–12.6, 17–193, 394.02–886.49, 130.06–511.04, and 24.5–41.62, and the mean values (mg·kg^−1^) were 76.84, 3.25, 99.76, 588.68, 284.21, and 34.71, respectively. The pH of the soil samples in the study area ranged from 7.5 to 8.6; according to the soil quality standard for agricultural land published by the Ministry of Ecology and Environment of China (GB 15618-2018), the concentrations of Cr and Pb were far less than the filter values, whereas Cd exceeded the control values in S3 (3.15 times) and the filter values in S1 (3.58 times), S4 (5.95 times), S5 (4.98 times), and S6 (1.42 times), suggesting Cd contamination around the HFR.

**Figure 4:**
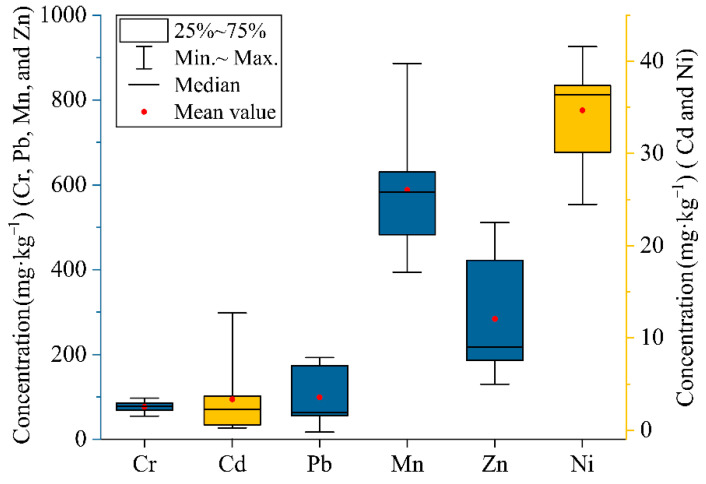
Boxplot of heavy metal concentration in the topsoil of the study area.

It is notable that the metal concentrations gradually decreased with increasing distance from the HFR, which differs from the results of some studies on wastewater irrigation areas [6]. As a result of the investigation, the crude irrigation method in the study area was historically pumped and flood irrigation was provided to the farmland over a long period: this was later changed to canal irrigation, both of which caused pollutants from the water source to radiate to the surrounding farmland. In addition, wheat and peanut are the main crops grown in the study area, and some studies have shown that legumes can absorb groundwater through capillary action to provide nutrients [66], while the root system of wheat reaches a depth of 1.5 m [67], which is undoubtedly beneficial to the growth of plants adjacent to the river. Groundwater elements also accumulate in the soil through the plants’ uptake. The box plot (Figure 4) visualizes Cr as having a narrow range of concentrations and, combined with the coefficient of variation (0.18%), Cr is found to show a stable spatial distribution in the study area, indicating that its concentration is unaffected by sewage irrigation, which matches the results of soil profile and sediment analysis.

### 3.4. Distribution of Heavy Metal Speciation in Sediment and Soil

Contrary to the total amount of heavy metals, the various speciation of heavy metals can characterize their release potential and toxicity in the environment [68], thus providing a better understanding of the extent of the impact of long-term sewage irrigation on agricultural soils. In this study, a modified BCR sequential extraction experiment was conducted on two sets of sediment profile samples and eight topsoil samples (0–100 mm, S1–S8), to better understand the historical concentration of heavy metals in river water and the risk of heavy metals being released from farmland soils in the SIA, respectively. The exchangeable fraction (F1), reducible fraction (F2), and oxidizable fraction (F3) represent the release capacity under normal, reducing, and oxidizing conditions, respectively.

**Figure 5:**
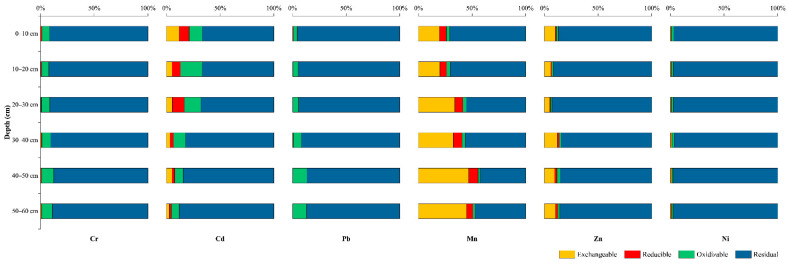
Percentage of each speciation for heavy metal in profile samples in HJR.

**Figure 6:**
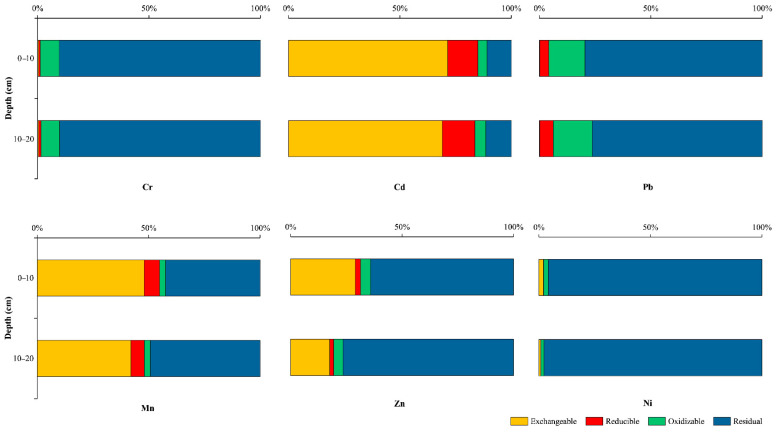
Percentage of each speciation for heavy metals in profile samples in HFR.

In the sediments, Cr, Pb, Zn, and Ni were observed to dominate the residual fraction (F4) in the HJR (Figure 5), with Zn accounting for an average of 9% in the F1, suggesting that these metals are not available under the aqueous environment in the HJR, while Zn has a slight release potential. Similarly, the F4 that is dominant in the HFR includes Cr, Pb, and Ni (Figure 6), while Zn has a higher F1 of 19%, on average, compared to the HJR. It is obvious that Cr and Mn have a high percentage of F1 in both sediment samples, especially in HFR, where the means of the sum of (F1 + F2 + F3) for Cr and Mn reach 88% and 50%, respectively, which indicates that they can facilitate molecular exchange with water and are readily absorbed by plants and animals.

**Figure 7:**
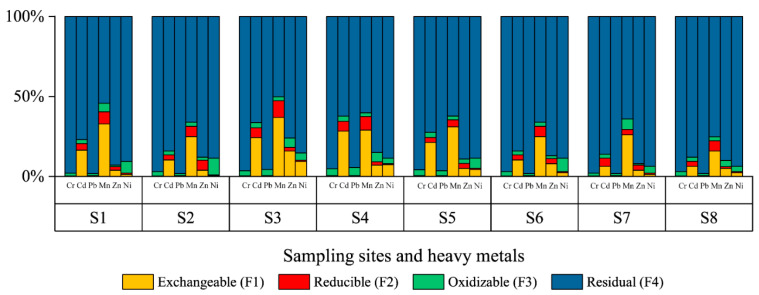
Percentage of each speciation for heavy metals in study area.

For the topsoils (Figure 7), as the residual fraction of heavy metals is mainly derived from the mineral and influenced by rock weathering and soil erosion, it is extremely difficult for them to migrate or be transformed in the environment, and thus Cr and Pb are the least toxic heavy metals in the study area and are not affected by sewage irrigation, which is consistent with the sediment findings. The average percentage of (F1 + F2 + F3) for Zn and Ni was 11%, and the most significant increase compared to the sediment was found in the Ni component (*p* < 0.01). It is possible that this change is the result of selective uptake by plants; Research on the absorption of specific heavy metals by various crops mentioned that wheat grown in sewage irrigation soil is enriched in Zn and Ni, especially Ni, which is the most likely heavy metal to be absorbed by plants, followed by Zn, Fe, Mn, and Cu, while wheat is the least effective at absorbing Mn among the numerous crops assessed [69]. Mn and Cd have the largest proportion of F1 exhibiting a particularly strong toxic potential at S3, and such extreme migration ability indicates that it comes from wastewater irrigation.

### 3.5. RAC

**Figure 8:**
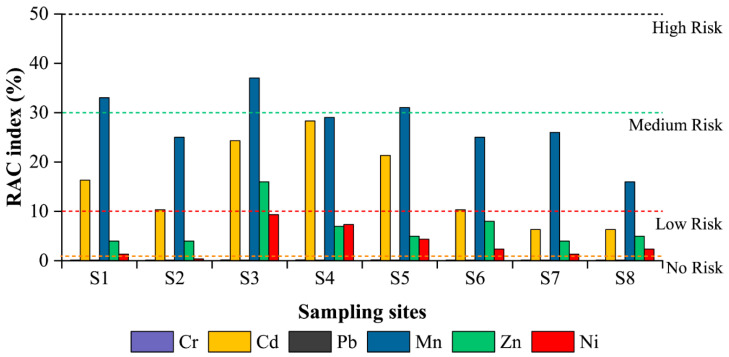
Level of RAC for heavy metals in study area.

The RAC quantifies the risk of releasing heavy metals in the environment based on the percentage of exchangeable form (F1) [49], and the RAC results for topsoil in this study are shown in Figure 8. Mn poses a high risk in S1, S3, and S5 while posing a moderate risk at other sites, while Cd poses a moderate risk from S1 to S6, indicating that Mn and Cd are the most available to transport to hazardous environments and enter the food chain in the study area. However, existing research on sediments and soils in non-contaminated irrigation areas indicates that Mn poses a higher risk than other heavy metals [48,70], and that the inherent chemical properties of Mn may determine its high mobility. Zn poses a low risk (except at S3), and Ni poses no risk at S2 and a low risk elsewhere. As discussed in the previous sections, the RAC results also showed that there is no risk posed by Cr and Pb.

For the sampling sites, S3 poses the greatest risk, followed by S4 and S5, while the control site (S8) was at minimal risk, except for that posed by Ni, which suggests that HFR contributes to the risk of soil contamination in the SIA.

### 3.6. The Effect of the Initial Concentration of Contaminants on Adsorption

**Figure 9:**
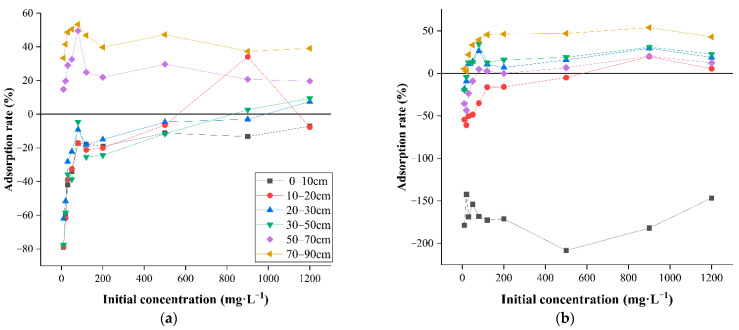
Changes in the adsorption rate of the soil within SIA for Cr (VI) with initial concentration ((**a**) is the soil profile sample of S1 and (**b**) is the soil profile sample of S2).

To further investigate the migration characteristics of soil heavy metals in the study area, two sets of profile samples, S1 and S2, with different heavy metal concentrations, were selected for isothermal adsorption experiments. Figure 9a,b separately show the changes in the adsorption rates of the soil from profiles at S1 and S2 for Cr (VI) with the initial concentration of Cr (VI). At a pH of 6, the influence of the initial concentration of Cr (VI) in the solution on the soil adsorption for Cr (VI) was explored. As shown in Figure 9, the adsorption rates of various soil layers in profiles for Cr (VI) increased with the increase in the initial concentration of Cr (VI). The reason for this was that it did not reach the maximum adsorption capacity of soil for Cr (VI) at a low Cr (VI) content. The adsorption rates of the soil layers at S1 were differentiated with the growth in the initial concentration of Cr (VI); except for the soil at depths from 500 to 900 mm, whose adsorption rate remained positive, the soil in various layers of the profile around HFR remained in a state of negative adsorption in terms of the adsorption rate for Cr (VI). For S2, the topsoil showed a significant negative rate of adsorption, while the adsorption rates in other layers gradually increased with depth and initial concentration, and all were positive at around 600 mg·L^−1^. The reason for this was that soil was polluted and the initial adsorption of soil for Cr (VI) reached a certain level, so the soil failed to continue its role as an adsorbent. As the initial concentration of Cr (VI) increased, the negative rate of adsorption constantly decreased, which indicated that the concentration of the prepared Cr (VI) solution could inversely inhibit the desorption effect of the soil. The desorption of soil was the inverse of the adsorption. The adsorption capacity of soil for Cr (VI) in an equilibrium state is important to assess the isolation of Cr (VI) in the unsaturated zone and desorption can influence the migration and transformation of Cr (VI) therein.

Adsorption capacity is one of the critical indicators when quantitatively assessing the adsorption effect of soil particles on heavy metals. The adsorption capacity of soil for Cr (VI) was calculated using Equation (3), and the amount of heavy metal adsorbed per unit of soil could be calculated by experimentally obtaining the reduction in adsorbed mass before and after the reaction. The isothermal sorption model was used to describe the Cr (VI) sorption process, explain the mechanism of Cr (VI) sorption on soils in the study area and calculate the maximum theoretical sorption capacity. The static adsorption of soil for Cr (VI) was simulated by using three isothermal adsorption models (i.e., Langmuir, Freundlich, and linear) [71,72,73], as separately given by Equations (4)–(6):

q_e_ = (C_0_ − C_e_) × V/m, (3)

q_e_ = (q_m_ × K_L_ × C_e_)/(1 + K_L_ × C_e_), (4)

q_e_ = K_F_ × C_e_^1/n^, (5)

q_e_ = K_d_ × C_e_, (6)

where q_e_, C_0_, C_e_, V, m, and q_m_ refer to the adsorption capacity (mg·kg^−1^) for Cr (VI), the initial concentration (mg·L^−1^) of Cr (VI), Cr (VI) concentration (mg·L^−1^) after reaction equilibrium, the volume (mL) of the initial solution, the mass (g) of the soil used, and the maximum adsorption capacity (mg·kg^−1^), respectively; K_L_ refers to a Langmuir constant related to the bonding strength, denoting the level of adsorption affinity of soil for heavy metals; K_F_ is a Freundlich adsorption constant, representing the adsorption capacity, related to the adsorbability (the larger the value, the larger the adsorption rate); 1/n denotes the empirical parameters pertaining during adsorption, which is generally less than 1 and more than 2 when the adsorption process is difficult to continue [74]; K_d_ is linear adsorption constants.

**Figure 10:**
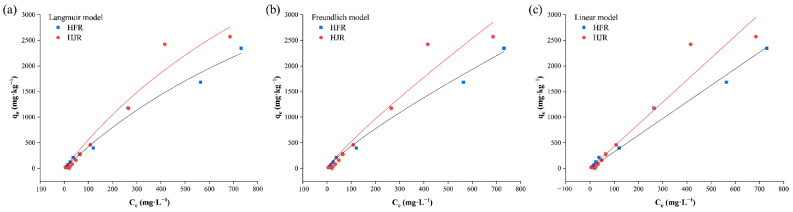
Models fitted to data related to adsorption of soil from profiles at depths of 700 to 900 mm within SIA for Cr (VI) ((**a**) is the curve fitted to the Langmuir equation, (**b**) is the curve fitted to the Freundlich equation, and (**c**) is the curve fitted to the Linear equation).

The adsorption characteristics of Cr (VI) in the soil at depths of from 700 to 900 mm around HFR (S1) and HJR (S2) were fitted by the three aforementioned isothermal adsorption models (Figure 10). Table 6 lists the related fitting parameters. For the isothermal adsorption tests of Cr (VI) in the soil, the three isothermal adsorption models all provided a good fit to the data related to the adsorption of Cr (VI) in soil within the unsaturated zone, in which the correlation coefficient reached a significant level (R^2^ > 0.9). The Langmuir isothermal adsorption model was a single-component adsorption model, and soil particles that fit this model are considered to be adsorbed in a single molecular layer and have a uniform distribution of ions adsorbed on the particle surface. As a result, the adsorption reaches saturation when all points are occupied, so the model was able to predict the maximum amount of Cr (VI) adsorption [75,76]. In contrast, the Freundlich model constructed an empirical formula for the adsorption behavior of multiple molecular layers, and the adsorption sites existed in multiple molecular layers, so it was not possible to calculate the maximum adsorption amount of soil particles that fit the model [74]. The 1/n values fitted using the Freundlich equation approximated to 1, implying a high adsorption reversibility, dominated by the distribution of adsorption [77,78]. The K_d_ values fitted using the linear equation were all low, and larger K_d_ values indicated a stronger adsorption capacity of the soil for Cr(VI). The adsorbability of the test soil around HJR for Cr (VI) was slightly stronger than that of the test soil around HFR. By contrast, the fitting result of soil adsorption for Cr (VI) using the Langmuir equation was superior to those obtained by applying the other two equations. The maximum adsorption capacities q_m_ of the test soils at HFR and HJR for Cr(VI) fitted using the Langmuir equation were 7012.198 and 8399.338 mg·kg^−1^, respectively; K_L_ representing the adsorption affinity of test soils at HFR and HJR for Cr (VI) was 0.0006 and 0.0007, respectively. The K_L_ value of the test soil around HJR was slightly larger than that around HFR, which agreed with the fitting result achieved using the linear equation. Based on the assumption of a Langmuir model, the results of isothermal adsorption experiments showed that the test soils had almost no affinity to Cr (VI), and the adsorption of Cr (VI) was characterized as a monolayer surface adsorption. The theoretical maximum adsorption capacity of the soil around HJR for Cr (VI) was more than that of HFR, which might be because the soil of HFR contained more heavy-metal ions in the previous heavy-metal contents analysis and occupied more adsorption sites on the particle surface.

## 4. Conclusions

The total concentrations and chemical speciation of heavy metals (Cr, Cd, Pb, Mn, Zn, and Ni) in soils and sediments were measured to determine the vertical and spatial distribution of heavy metals and assess the ecological risk of the release thereof. The main findings of the research are as follows: (1) Heavy metals in soil and sediment show different distributions in their profiles, and the concentrations of heavy metals in sediment gradually increased with depth, showing the historic severe pollution in river water; (2) For most sampling sites in the study area, Cd and Mn were the most contaminated, with Cd (12.6 mg·kg^−1^) exceeding the agricultural land control value by a factor of 3.15 near HFR, while the average concentration of Mn reached 588.68 mg·kg^−1^, but remained acceptable compared to other studies; (3) The results of RAC also indicated that Cd and Mn were the most ecologically risky factors, with both reaching moderate risk, where Mn posed a high risk in S1, S3, and S5 near the HFR. The distribution of heavy metals in sediment, topsoil, and the RAC results implied that HFR has a higher contamination level compared to HJR, which was related to the multiple industrial plants in the vicinity of HFR. Cr and Pb posed the lowest risk factors in the study area and the RAC posed no risk; (4) As they are easily soluble and readily absorbed by crops, the results of isothermal sorption experiments on Cr (VI) showed that the soil in the study area under long-term sewage irrigation conditions had a great capacity for Cr (VI) sorption. The soil samples near HFR found that the positive adsorption rate for Cr was sustained at 500–700 mm, while the rate of adsorption of the soil near HJR gradually shifted from negative to positive with increasing depth, except between 0–100 mm. Isothermal adsorption experiments showed that the process of Cr (VI) sorption by soils in the study area could be described using the Langmuir model, meaning single-molecule layer sorption at the surface layer of soil particles. Although this adsorption capacity is limited, the soil around HJR was slightly higher than HFR in terms of Cr adsorption capacity, and the introduction of Cr-containing wastewater into the area should be avoided.

In the future, changes in the Cd content in the study area should be noted to prevent excessive enrichment in crops and, thus, entry into the food chain. In addition, sediment should be a noteworthy objective for effluent pollutant carriers in SIAs, where long-term heavy metal enrichment may lead to the release of heavy metals, thus causing hazards to the aquatic environment.

## Figures and Tables

**Table 1:** Modified BCR sequential extraction scheme used for heavy metal speciation.

Step	Fraction	Extractant	Shaking Time and Temperature
F1	Exchangeable	32 mL of 0.11 M CH_3_COOH	End-over-end shaking at 22 ± 5 °C for 16 h
F2	Reducible	32 mL of 0.5 M NH_3_OH·HCl (pH 1.5)	End-over-end shaking at 22 ± 5 °C for 16 h
F3	Oxidizable	8 mL of 8.8 M H_2_O_2_ (pH 2)	Manual occasional shaking at 22 ± 5 °C for 1 h, then conducting water bath evaporation to 2 mL residue at 85 ± 2 °C
		8 mL of 8.8 M H_2_O_2_ (pH 2) was added again	Continuing water bath evaporation to 2 mL residue at 85 ± 2 °C
		Cooling was completed, and 32 mL of NH_4_OAc (pH 2) was added	End-over-end shaking at 22 ± 5 °C for 16 h
F4	Residual	0.2 g air-dried residue was taken	Same digestion scheme as pseudo-total heavy metals

**Table 2:** Corresponding contamination risk level by RAC.

RAC Index (%)	Risk Level
<1	No risk
1–10	Low risk
11–30	Medium risk
31–50	High risk
>50	Very high risk

Classification sources: The study of Perin et al. [49]. Reprinted/adapted with permission from Ref. [49]. 1985, Guido Perin.

**Table 3:** Concentrations (mg·kg^−1^) of heavy metals in profiled soils.

Site	Cr				Cd				Pb				Mn				Zn				Ni			
	Max	Min	Mean	SD	Max	Min	Mean	SD	Max	Min	Mean	SD	Max	Min	Mean	SD	Max	Min	Mean	SD	Max	Min	Mean	SD
S1	69	47.7	59.43	8.48	2.24	0.12	1.14	0.82	59.9	18.1	36.38	17.2	952.46	451.46	672.25	191.3	256.68	62.18	110.18	69	32.13	16.46	22.23	5.5
S2	80	58.6	69.77	7.6	0.46	0.16	0.31	0.12	64.3	22.1	41.77	18	612.65	442.53	503.69	60.5	184.05	66.2	92.2	41.8	25.75	17.28	21.33	2.74
S8	91.1	73.4	85.35	5.7	0.23	0.12	0.15	0.04	30.7	18.8	25.2	3.74	483.41	434.18	458.66	18.2	97.81	62.67	75.39	12.5	20.11	16.49	18.14	1.09

**Table 4:** Heavy-metal concentration (mg·kg^−1^) in surface sediments.

Heavy Metal	Cr	Cd	Pb	Mn	Zn	Ni	Reference
HFR	87.79	13.73	224.49	570.6	566.31	53.89	This study
HJR	74.93	0.6	101.78	363.18	187.25	41.96	This study
Mighan Lake	29	N/V	12.43	N/V	174	17.43	[62]
Masan Bay	N/V	13	86	N/V	N/V	N/V	[63]
Beitang River	N/V	1.73	289.1	N/V	N/V	N/V	[64]
Guideline value	26	0.6	31	N/V	120	16	[65]

N/V = no value derived.

**Table 5:** Heavy-metal concentration (mg·kg^−1^) in topsoil.

Heavy Metal	Cr	Cd	Pb	Mn	Zn	Ni
S1	68.7	2.15	58.3	886.49	217.56	30.15
S2	77.75	0.45	62.9	583.64	130.06	24.5
S3	85.5	12.6	193	625.15	511.04	41.62
S4	97.2	3.57	174	630.18	422.1	37.41
S5	84	2.99	137	518.7	314.92	36.4
S6	69.8	0.85	56.1	482.6	207.62	35.77
S7	54.9	0.12	17	394.02	186.13	37.1
Mean	76.84	3.25	99.76	588.68	284.21	34.71
C.V. (%)	0.18	1.33	0.68	0.26	0.49	0.16
Filter value ^1^	250	0.6	170	N/V	N/V	N/V
Control value ^2^	1300	4	1000	N/V	N/V	N/V

N/V = no value derived. ^1^ Below the filter value indicating low risk of soil contamination in agricultural land. ^2^ Higher than filter value and less than or equal to control value, denoting that food agricultural products do not meet quality and safety standards and other soil contamination risks; above control value, meaning that food agricultural products do not meet quality and safety standards and the risk of soil contamination on agricultural land is high.

**Table 6:** Fitting parameters of isothermal adsorption models of soil from the profile at depths of 700 to 900 mm within SIA for Cr (VI).

Source of Soil	Langmuir Equation			Freundlich Equation			Linear Equation	
	*q_m_*	*K_L_*	*R* ^2^	*K_F_*	1/*n*	*R* ^2^	*K_d_*	*R* ^2^
HFR	7012.198	0.0006	0.984	9.446	0.832	0.984	3.240	0.976
HJR	8399.338	0.0007	0.950	9.225	0.878	0.937	4.306	0.937

## Data Availability

Not applicable.
